# Effect of Glycine Betaine on Chilling Injury in Eggplant Peel Revealed by FTIR Spectroscopy

**DOI:** 10.3390/foods15101704

**Published:** 2026-05-13

**Authors:** Michelle Guijarro-Fuertes, María José Zaro, Analía Concellón, Gemma Montalvo, Fernando Ortega, Armando Echeverría, Pablo Moncayo, Luis Ramos-Guerrero

**Affiliations:** 1 Centro de Investigación y Desarrollo en Ciencia y Tecnología de Alimentos (CIDCA), CONICET, Facultad de Ciencias Exactas, Universidad Nacional de La Plata, La Plata 1900, Argentina; michelleguijarro@quimica.unlp.edu.ar (M.G.-F.); maria.zaro@agro.unlp.edu.ar (M.J.Z.); aconcell@quimica.unlp.edu.ar (A.C.); 2 Universidad de Alcalá, Grupo CINQUIFOR, Departamento de Química Analítica, Química Física e Ingeniería Química, Ctra. Madrid-Barcelona km 33.6, 28871 Alcalá de Henares, Madrid, Spain; gemma.montalvo@uah.es; 3 Universidad de Alcalá, Grupos CINQUIFOR and HCIS, Departamento de Ciencias de la Computación, Química Física e Ingeniería Química, Ctra. Madrid-Barcelona km 33.6, 28871 Alcalá Madrid, de Henares, Spain; fernando.ortega@uah.es; 4 Unidad Académica para la Formación Técnica y Tecnológica, Universidad Católica Santiago de Guayaquil, Guayaquil 090615, Ecuador; neptali.echeverria@cu.ucsg.edu.ec; 5 Facultad de Ingeniería y Ciencias Aplicadas, Carrera de Ingeniería Agroindustrial, Universidad de Las Américas (UDLA), Quito 170124, Ecuador; pablo.moncayo@udla.edu.ec; 6 Facultad de Ingeniería y Ciencias Aplicadas, Carrera de Ingeniería Agroindustrial, Grupo de Investigación en Alimentos y Agroindustria (GIAA), Universidad de Las Américas (UDLA), Quito 170124, Ecuador

**Keywords:** *Solanum melongena* L., maturity stage, FTIR Spectroscopy, chemometrics, postharvest treatment, rigidity

## Abstract

Eggplant (*Solanum melongena* L.) is highly susceptible to chilling injury (CI) during cold storage, with severity being strongly influenced by fruit maturity stage. At the tissue level, the peel acts as the primary site of cold-stress metabolic responses. This study evaluated the effect of pre-storage glycine betaine treatment (GB, 10 mM) on CI in purple eggplant at baby (BB, low sensitivity) and commercial (CC, high sensitivity) maturity stages stored at 4 °C for 20 days, integrating the use of ATR-FTIR spectroscopy as a rapid, non-destructive tool to monitor quality in the fruit peel. Physiological traits including chilling injury index (CII) and fruit rigidity were integrated with peel-specific ATR-FTIR spectroscopy combined with chemometric analysis to describe structural and metabolic behavior. BB fruit showed higher tolerance to CI, reaching a CII 23% lower than CC after 20 d, along with greater rigidity retention. GB treatment was significantly effective in reducing CI in both maturity stages by decreasing CII by 23% for BB and 32% for CC fruit, and delaying symptom onset. FTIR analysis revealed that the main peel spectral changes during storage occurred in the amide–phenolic (1653–1515 cm^−1^) and polysaccharide (~1017 cm^−1^) regions. Control fruit showed progressive shifts in these regions indicating structural disorganization, while GB-treated fruit delayed and attenuated spectral changes. Chemometric analysis (OPLS-DA) clearly discriminated samples according to maturity stage, treatment, and storage time. Overall, the results demonstrate that chilling susceptibility is determined by maturity stage, that the GB treatment enhanced CI tolerance—especially in sensitive CC fruit—and that ATR-FTIR coupled with chemometrics provides an effective approach for rapid non-destructive monitoring of postharvest quality changes in eggplant during cold storage.

## 1. Introduction

The fruit and vegetable supply chain represents 20–50% of the food losses, due to their high moisture content and perishable nature [1,2,3]. Even though refrigeration is considered a key element in maintaining the postharvest quality of fresh products, fruits such as eggplants are prone to chilling injury (CI) under low-temperature storage (<10 °C) due to their cold-sensitive condition [4,5]. The eggplant fruit is well known for its low-fat content, fiber, protein, vitamins and minerals contribution in addition to its antioxidant contents, especially anthocyanin in the peel [6].

In sensitive vegetables, CI varies according to the variety, tissue or maturity stage. Studies report high cold sensitivity at immature stages rather than their mature ones in tomato, cucumber and bell pepper [7,8,9,10]. Notably, eggplant showed more serious chilling damage at later maturity stages compared to early stages, demonstrating an opposite trend from the other *Solanaceae* [11,12]. The biochemical mechanisms underlying this unusual behavior are still under study. CI in eggplant affects both microscopic and macroscopic levels. CI manifests as fruit dehydration, peel pitting and scalds, and pulp browning, leading to a deterioration in appearance, nutritional and physical quality. At the cellular level, membrane integrity is compromised, inducing lipid phase transitions, lipid peroxidation, and the accumulation of reactive oxygen species (ROS). These alterations disrupt cellular energy homeostasis, ultimately resulting in programmed cell death and the subsequent tissue collapse associated with structural degradation [12]. Moreover, peel deterioration constitutes a critical postharvest limitation from a commercial standpoint, since visual quality strongly determines consumer acceptance and purchasing decisions. Understanding the biochemical and structural changes occurring in this tissue is therefore essential to design strategies aimed at delaying the onset and progression of CI symptoms. In this regard, in a previous work it was reported that eggplant tissues showed a specific metabolic response to cold stress, with the peel more reactive than the flesh, including accumulation of compatible solutes and unsaturated fatty acids [13]. Several pre-storage treatments were applied to horticultural products to reduce the CI impact in storage at low temperature, such as heat application, atmosphere modification, or UV radiation [14,15,16,17,18]. The use of chemical treatments has also gained interest owing to the use of natural products such as plant hormones and natural elicitors like glycine betaine (GB) [19]. This compound is characterized by its high hydrophilicity and its origin within the chloroplasts, where it serves the essential function of maintaining cellular homeostasis through osmoregulation. Therefore, GB acts like pigments and cell membrane stabilizer [19]. Consequently, treatments with GB are frequently applied to fruits like bananas and pomegranates [20], as well as vegetables like zucchinis, prior to cold storage [21]. In addition, studies report that GB treatment delays the incidence of CI, such as pitting in hawthorn peel, pulp browning in pear fruit, or enhances the physiological response to cold storage in zucchini [21,22,23]. In commercially mature eggplants, treatment with 10 mM glycine betaine (GB) significantly reduced CI-related indicators, such as weight loss, respiration rate, electrolyte leakage and malondialdehyde content and extended shelf life by maintaining fruit firmness and antioxidant capacity [13]. However, the effectiveness of GB in preserving the quality of cold-stored baby eggplants (early stage with less sensitivity to CI) has not yet been investigated. This could help to understand whether GB acts uniformly or differentially in systems with different sensitivity to chilling injury.

Biochemical and metabolic changes preceding visible CI symptoms are difficult to assess due to labor-intensive sample preparation, time-consuming assays, and the use of large solvent volumes that generate environmental waste. Hence, there is a growing need for non-destructive and simple analytical tools during postharvest handling [24]. Spectroscopic techniques provide qualitative and quantitative information from various matrices with minimal preparation. These methods rely on light–sample interactions that generate spectral data, expressed as absorption peaks corresponding to chemical bonds and functional groups [25,26,27]. Combined with chemometric models, spectroscopy supports decision-making in postharvest management, enabling rapid quality assessment and by-product valorization. Applications in apricots and berries have demonstrated efficiency in monitoring quality attributes and antioxidant profiles during storage, reducing time and costs [28,29]. It has also been applied to evaluate polyphenolic extracts and cell wall composition in different fruits and vegetables like red pepper [30], red black mulberry, papaya, banana peel, sweet potato, squash and other fruits [27]. Furthermore, ATR-FTIR spectroscopy has been used to discriminate metabolic profiles in banana accessions, revealing variability in carbohydrate, protein, and lipid regions [31].

This study aimed to assess the effect of pre-storage glycine betaine treatment (GB) on CI control in purple eggplant at baby (BB, low sensitivity) and commercial (CC, high sensitivity) maturity stages during cold storage, integrating the use of ATR-FTIR spectroscopy as a rapid, non-destructive tool to monitor quality in the fruit peel.

## 2. Materials and Methods

### 2.1. Samples, Treatment, and Storage Conditions

Purple eggplant (*Solanum melongena* L.) fruits produced in a greenhouse of La Plata city (Buenos Aires, Argentina) were hand-harvested at baby (BB, 9 cm) and commercial (CC, 17 cm) maturity stages employing an average length as harvest index [12]. The fruits were transported immediately to the laboratory and carefully selected, washed with sodium hypochlorite solution (200 mg L^−1^), and air-dried at room temperature. Each maturity stage group was randomly divided into control (CT) and treated with glycine betaine (GB) groups, with fifty fruits each.

The CT group was immersed for 10 min in distilled water and Tween (0.1%); meanwhile, for the treated group, 10 mM glycine betaine (GB) aqueous solution (trimethylglycine, Sigma B2629, St. Louis, MO, USA) with Tween (0.1%) was used. The 10 mM GB concentration was selected based on our previous study [13], reporting its effectiveness in commercial purple eggplant in comparison to other concentrations. The fruits were air-dried, packed into plastic trays, and covered with perforated PVC film. The trays were stored for 20 days at 4 °C and 90% relative humidity. On each sampling day (0, 8, 15, and 20), at least twelve eggplant fruits from both groups, CT and GB, were analyzed for chilling injury index (CII) and rigidity. Then, the peel tissue was removed with a scalpel, recollected and immediately frozen in liquid nitrogen, freeze-dried, and stored in a desiccator until used for FTIR determinations.

### 2.2. Determination of Quality Parameters

- Chilling Injury Index: Twelve fruits were collected at each sampling day of every maturity stage to assess CII. The levels of the scale to define CII were based on the extension of peel with pitting, scald or visible browning related to cold stress, where 1 represents no injury, and 2, 3, 4, 5 show 1–25, 26–50, 51–75, >76% of surface with injury symptoms, respectively. The CII was calculated using the formula according to Massolo [32]:
  $$CII=\frac{\;\left(1\times Number\;of\;fruits\;in\;level\right)+\left(2\times Number\;of\;fruits\;in\;level\right)+\ldots \left(5\times Number\;of\;fruits\;in\;level\right)}{Number\;of\;total\;fruits}$$
- Rigidity (S_i_): Eggplant fruits were compressed with a 3 mm probe for 8 mm distance at the equatorial position at a 1 mm s^−1^ rate using texture equipment (TA.XT2, Stable Micro Systems, USA). The results were expressed in N mm^−1^. Nine fruits of each maturity stage, treatment and storage day were used, and three measurements were done on opposite sides of each fruit. The initial slope (S_i_) of the curve represents the force after two and four seconds at a speed of one second. The slope calculation was based on the following equation [33]:

$$S_{i}=\frac{\left(F2-F1\right)}{Distance\;2-Distance\;1}$$

where F2 and F1 were the force (N) at distance 2 (4 mm) and distance 1 (2 mm), respectively.

### 2.3. FTIR Spectral Characterization

A fragment of the freeze-dried peel from the CT and GB fruits from each sampling date was analyzed by FTIR. The infrared spectral measurements were obtained using a Cary 630 FTIR spectrometer (Agilent, Santa Clara, CA, USA) equipped with a ZnSe crystal and KBr optics, controlled by the MicroLab FTIR Software v5.8 (Agilent, Santa Clara, CA, USA). All spectra were collected by co-adding 64 scans at a resolution of 4 cm^−1^ in the range of 4000–600 cm^−1^ on the twelve fragments from each maturity stage, treatment, and storage conditions. Each spectrum was ratioed against a background spectrum obtained every 15 min.

### 2.4. Statistical Design

CII and rigidity data were analyzed with ANOVA using the InfoStat software v2020, and means were compared using the Fisher test at a significance level of α = 0.05.

Prior to the chemometric analysis, the entire collected spectra were pre-processed using standard normal variate (SNV) normalization, baseline correction with asymmetric least squares (AsLS), and smoothing with the Savitzky–Golay method. The multivariate data analysis was performed using the orthogonal partial least squares discriminant analysis (OPLS-DA) procedure within SIMCA v18 (Sartorius Stedim, Göttingen, Germany). OPLS-DA is a blend of supervised PLS regression and orthogonal signal correction (OSC) and then combined with discrimination analysis (DA). In this form of modeling, the information in the X-data matrix is divided into two blocks: one correlated to Y, and another non-correlated (orthogonal) to Y. The models were automatically cross-validated by SIMCA’s built-in leave-one-out method. To do so, the data were divided into seven groups. For the first cross-validation (CV), one of the groups was automatically excluded; the model was made with the other six groups, and the excluded group was used to test the model. Then, the excluded group was included, and a new group was excluded, and the process was repeated with all seven groups. This CV was done to avoid under- or over-fitting of the final OPLS-DA model.

## 3. Results and Discussion

### 3.1. Quality of Baby and Commercial Eggplants and FTIR Spectroscopy Peel Characterization at Harvest

**Figure 1:**
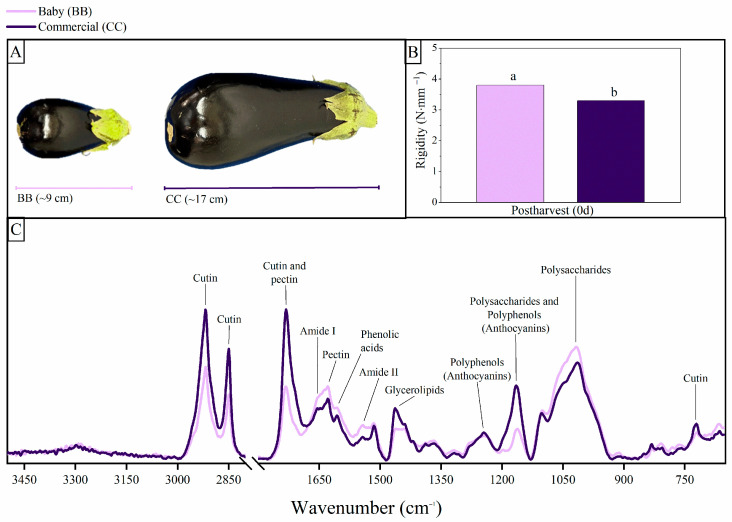
(**A**) Appearance and lengths, (**B**) rigidity where different letters indicate significant differences between samples (*p* < 0.05) and (**C**) FTIR spectra of purple eggplant fruits harvested at baby (BB) and commercial (CC) maturity stages according to references listed in Table 1.

At harvest, baby (BB, 9 cm) and commercial (CC, 17 cm) eggplants showed distinct appearance, physical and spectral characteristics (Figure 1). The calyx and the characteristic intense purple coloration of the peel are already visible from the early stages of the fruit development (Figure 1A). As growth progresses, the fruit elongates and reaches sizes commercially acceptable, although small or baby eggplants are also in high demand in the gourmet market. The rigidity for the BB and CC fruit stages was 3.8 and 3.3 N mm^−1^, respectively (Figure 1B). These results agree with our previous report where eggplants softened markedly in transition from preliminary ontogeny baby stage to full-sized fruit [12]. The higher firmness observed in BB eggplants can be attributed to several structural and physiological factors. According to Valerga [34], the BB fruits possess turgid cells with full vacuoles that enhance compression resistance. Additionally, the parenchyma at this stage has fewer intercellular spaces and is more compact with respect to CC fruits, which showed increased air spaces.

Figure 1C shows the average ATR-FTIR spectra of the BB and CC eggplant peels at harvest, covering the 4000–600 cm^−1^ region. Several intense absorption bands were observed in three main regions corresponding to fundamental transitions: the X–H stretching (4000–2800 cm^−1^), the double-bond (2000–1500 cm^−1^), and the fingerprint (1500–600 cm^−1^) regions (Figure 1C and Table 1). The spectra showed similar band positions in both stages, but clear differences in band intensities. The most relevant variations were observed at 2919 and 2850 cm^−1^ (aliphatic methyl groups related to cutin), 1730 cm^−1^ (ester carbonyl related to cutin and pectin), 1653–1515 cm^−1^ (Amide I and II related to protein and phenolic backbone), 1163 cm^−1^ (polysaccharides and flavonoids), and ~1017 cm^−1^ (polysaccharides). BB fruit showed higher relative intensity in phenolic- and polysaccharide-associated regions, whereas CC fruit exhibited stronger signals in cutin and ester-related bands (Supplementary Material). Overall, these results indicate that maturity stage defines the initial structural organization of the peel, which may influence subsequent responses to chilling stress.

Notably, Table 1 compiles, for the first time, the principal FTIR bands identified in eggplant peel at two contrasting stages of maturity, together with their proposed compound assignments, providing a concise framework for interpreting the spectral features described in this study. Histologically, the eggplant peel consists of cuticular and epidermal layers, with a hypodermal layer also present in some *Solanaceae* species. The cuticular layer is mainly composed of cutin and epicuticular or intracuticular waxes, which form a hydrophobic barrier. In purple eggplant fruits, the epidermis is 1–4 µm thick and contains pigments such as anthocyanins [34,35,36,37]. In the following sections, the BB and CC peel systems will present the characteristic signals of these compounds.

Cutin is an apoplastic, insoluble polyester generally composed of oxygenated fatty acids (C16 and C18), glycerol and phenolic compounds such as hydroxycinnamic acids and flavonoids [38,39,40]. The FTIR signals at 2919 and 2850 cm^−1^ can be attributed to the asymmetric and symmetric vibrational modes of aliphatic methyl groups (Table 1) [41,42,43,44,45]. Moreover, the signal at 1730 cm^−1^ corresponds to the carbonyl (C=O) stretching vibration of ester bonds (Table 1) [41,46,47]. These bands are considered characteristic of cutin polyester [30]. In addition, the asymmetric vibration of C–O–C esters in the cutin matrix generates a strong signal in the fingerprint region at 1165 cm^−1^. The intensity of these signals (2919, 2850, 1730, 1165 cm^−1^) marked a clear difference here in the peel eggplant tissue, being more intense in the CC than in BB (Figure 1C). Similar trends were found in tomatoes, in which cutin monomers increased throughout their development and may be related to the mechanical properties of the fruit [38]. According to the ratio of C16/C18 cutin monomers, cuticles dominated by C16 are more rigid, whereas near-equal mixtures are more elastic [38,40,48,49].

The epicuticular wax in eggplant peel has been reported to be dominated by n-alkanes and branched alkanes, as well as aldehydes, n-alkanoic acids, and triterpenoids, including sterols [50]. These compounds generate a characteristic signal at 1463 cm^−1^ (scissoring of CH_2_ vibration mode of long aliphatic chains) (Table 1) [30,41,47,50]. This signal overlaps with cutin but is stronger in CC than in BB peel, reflecting a progressive accumulation of waxes during development. This trend aligns with ripening studies in tomatoes, which demonstrated an increase in wax layer thickness [51].

Chlorogenic acid is the main phenolic compound in eggplant peel and pulp, formed from esterification of caffeic and quinic acids [52]. A previous work reported that the eggplant fruits at early ontogenetic stages presented higher levels of this compound compared with late mature stages [52]. The backbone of phenolic compounds (C-C aromatic) exhibits a characteristic band at 1515 cm^−1^ (Table 1 and Supplementary Material) [27,41,53], which was more intense in BB than in CC fruit (Figure 1C). In addition, studies have shown that mechanical performance can also be regulated by the amount of phenolic acids in the cuticle. In isolated tomato cuticle, it was observed that early stages characterized by low phenolic content presented a soft, elastic behavior, whereas the phenolic accumulation during ripening was associated with cuticle strength [54]. In this regard, in eggplant, the higher spectral intensity in the phenolic-associated region of the peel at the BB stage could contribute to the higher fruit firmness, continuing to show differences in performance between immature and mature stages compared to other *Solanaceae*.

The polysaccharides in eggplant peel are mainly cellulose, consistent with the high content of insoluble dietary fiber. Spectroscopically, cellulose and hemicellulose exhibit characteristic bands in the 1020–1032 cm^−1^ region, attributed to C–O and C–C vibrations (Table 1) [25,27,30,55]. Pectin, an acidic heteropolysaccharide, was also identified, with its presence linked to very strong signals at 1731 and 1014 cm^−1^ (Table 1) [25,30,41,45,46]. These would correspond to the C=O stretching vibration of esterified galacturonic acids, as well as C–O and C–C stretching and backbone vibrations (C2–C3, C2–O2, C1–O1) [27,55]. Moreover, the very strong absorption bands at 1074, 1600–1632, and 831 cm^−1^, arising from C–C ring stretching, COO^−^ antisymmetric stretching, free carboxyl groups, and ring vibrations, respectively, are associated with the rhamnogalacturonan I and II, homogalacturonan, and polygalacturonic acid structures (Table 1) [47,55,56].

The spectral profiles revealed distinct differences between the samples. Specifically, the signals at 1600–1632 cm^−1^ and 1014 cm^−1^ were more intense in BB than in CC peel. In the 1600–1632 cm^−1^ region, the signal intensity in BB suggests a predominant protein component, likely due to an overlap with the Amide I band. Conversely, in the CC stage, this band appeared broader or shifted toward 1600 cm^−1^, a characteristic associated with the accumulation of de-esterified pectins (Figure 1C). Furthermore, the spectra clearly illustrated the influence of methoxylation on the signal intensity at 1731 cm^−1^. While the BB stage demonstrated a higher relative cellulose content, the CC stage exhibited a greater contribution from soluble pectins. These compounds also determine mechanical properties. Polysaccharides contribute to linear elasticity, high modulus, and breaking stress, while cutin provides viscoelasticity and extensibility. Consistent with this, most bands were more intense in BB than CC, distinguishing maturity stages [54,57,58]. Notably, the very strong intensity of the 1730 cm^−1^ band in CC peel eggplant fruits, compared to the BB peel, would be related to a high degree of methylation (Figure 1C and Table 1). However, in the BB peel, the more intense signal at 1606 cm^−1^, relative to CC, is associated with non-esterified polygalacturonic acid. Additional studies have shown that pectin content increased during eggplant development because of new cell wall deposition [12,56].

**Table 1:** Experimental frequencies (cm^−1^) and assignment of the FTIR spectra average for the peel of baby and commercial purple eggplant.

Experimental Wavenumber (cm^−1^) and Peak Characteristics		Reported Wavenumber (cm^−1^)	Assignment Vibrational Modes	Proposed Compound	Reference
Baby	Commercial				
~3297 (w-broad)	~3299 (w-broad)	~3400 (broad)	ν (O-H…O) of hydroxyl groups	Polysaccharides, cutin, and phenolic and anthocyanins compounds	[41]
2960 (m-shoulder)	2958 (m-shoulder)	2956 (shoulder)	ν_as_(C–H) of aliphatic CH_3_ group and steroidal structures	Cutin and steroidal alkaloids	[59,60]
2917 (s)	2919 (vs)	2920 (s)	ν_as_ (C–H) of aliphatic CH_2_ and CH_3_ groups and steroidal structures	Cutin and steroidal alkaloids	[30,41,42,44]
2848 (s)	2850 (vs)	2804, 2850 (s)	ν_s_(C–H_2_)	Cutin and steroidal alkaloids	[45]
1729 (m)	1731 (vs)	1730 (s)–1750	ν(C=O) of esters	Cutin, pectin and alkaloids	[45,46,61]
1709 (m-shoulder)	1711 (s-shoulder)	1715	ν(C=O) of esters; carboxylate ion stretching –COO–	Cutin, phenolic compounds	[41,46]
1653 (m)	1655 (m)	1650–1653	Bivalent C-bonds ν(C=O, C-N) of Amide I δ(N-H) pyrrolidine ring	Protein and glycoalkaloids	[42,62]
1631 (s)	1629 (m)	1628	ν(C=C) of phenolic acid ν_a_(COO-)	Phenolic acids Non-esterified pectin	[27,47,63]
1606 (m)	1606 (w)	1603	ν(C-C) aromatic ν(COO-); ν_s_ aryl-ring	Non-esterified pectin; phenolic compounds	[41,42,47]
1554 (w)	1560 (w)	1551 (w)	ν(C-C) aromatic conjugated with C=C	Phenolic and anthocyanin compounds	[25,41,47]
1539 (m)	1541 (m)	1540	δ(C-N); ν(C-N) of amide II and steroidal alkaloids	Protein and glykoalkaloids	[42,63,64]
1515 (m)	1515 (m)	1513	ν(C-C) aromatic conjugated with C=C	Lignin and phenolic backbone	[27,41,51]
1463 (m)	1463 (m)	1460–1473	δ(CH_2_) scissoring	Cutin, glycerolipids, wax hydrocarbons, steroidal rings	[30,41,47,51]
1457(m-shoulder)	1457 (m)	1451–1456	δ(CH_2_)	Cellulose polysaccharides	[42,65,66]
1439 (m)	1439 (w)	1434–1439	δ(CH_2_) ν(O-CH_3_) ν(C-C) of aromatic skeleton conjugated with C=C	Polysaccharides and cutin Flavonoids (anthocyanins)	[42,66]
1418 (w)	1418 (w)	1410, 1426	ν(COO-)	Pectin and polysaccharides	[27,42]
1388 (w)	1388 (w)	1377	δ(CH_3_)	Polysaccharides	[42,51,66,67]
1364 (w)	1366 (w)	1346	δ(CH_2_) wagging and twisting	Cutin	[41,68]
1278 (m-shoulder)	1280 (m-shoulder)	1280	ν(C-O-C)	Lipids	[41,42,69]
1258 (m-shoulder)	1265 (m-shoulder)	1265	ν (O-C); ρ_as_(CCO); ρ(OH)	Flavonoid compounds (anthocyanins)	[62,70]
1243 (m)	1245 (m)	1250–1246	ν((heterocycle); ν(C–OH)	Flavonoid compounds (anthocyanins)	[71]
1193 (m-shoulder)	1196 (s-shoulder)	1198	δ(OH)	Cellulose	[42]
1163 (m)	1165 (vs)	1160–1165	ν_as_(C-O-C) of ester and glycosidic bonds	Cutin, flavonoid compounds (anthocyanins) and glycoalkaloids	[42,47,67,72]
1105 (s)	1103 (s)	1105	ν_s_(C-O-C) of ester Phenol δ(C–O–C) pyranose ring	Cutin Polysaccharides	[60,63,73]
1077 (s-shoulder)	-	1078	ν (C–O); ν (C–C) ν (C–OH) ν_s_(PO2)	Xyloglucan Oligosaccharide Phospate	[47]
1068 (s)	1070 (s-shoulder)	1060	ν (C-O); ν (C-C)	Polysaccharides and glycosidic structures	[42,46,74]
1053 (vs)	1049 (s)	1050	ν(C–O-C) glycosidic bonds	Polysaccharides and flavonoid compounds (anthocyanins)	[41,42,75]
1027 (vs)	1028	1020–1032	ν(C–O) ν(C–C)	Cellulose Hemicellulose	[25,27,30,55]
1018 (vs)	1016 (vs)	1018 (s)	ν(C-O); ν(C-C); ν(C-O-H)	Polysaccharides, pectin	[25,30]
1008 (vs-shoulder)	-	1003 (w)	ν(C-O, C-C) δ(O-C-H)	Homogalacturonan	[41,76]
967 (s-shoulder)	967 (s-shoulder)	967 (m-shoulder)	ν(C–O) ρ(C-O-C)	Polysaccharides Glycosidic structures	[41,45]
989 (w-shoulder)	990 (w-shoulder)	991–995	ν(C–O) ν(C–C)	Cellulose	[27,41,56,66]
909 (w)	911 (w)	897–903	β(1–4) gl	Cellulose, hemicellulose	[77]
831 (w)	831 (w)	832	γ(C-H) aromatic	Pectin and phenolic compounds	[25,41,55,56]
806 (w)	806 (w)	811–818	γ(C-H)	Hemicellulose and phenolic compounds	[42,56]
719 (w)	720 (w)	722	δ(C-H_2_ rocking) and H_2_ groups with bivalent C-bond	Cutin	[42,67,72]
663 (w)	663 (w)	663–668	γ(C-O-H)	Cellulose	[42]

Vibrational modes: δ (bending), ν (stretching), ν_as_ (asymmetric vibration), ν_s_ (symmetric vibration), δ (scissors), γ (out-of-plane bending), ρ (rocking). Intensity: w (weak), m (medium), s (strong), vs (very strong).

### 3.2. Effects of Glycine Betaine Treatment on Chilling Injury Development During Cold Storage of Eggplant

**Figure 2:**
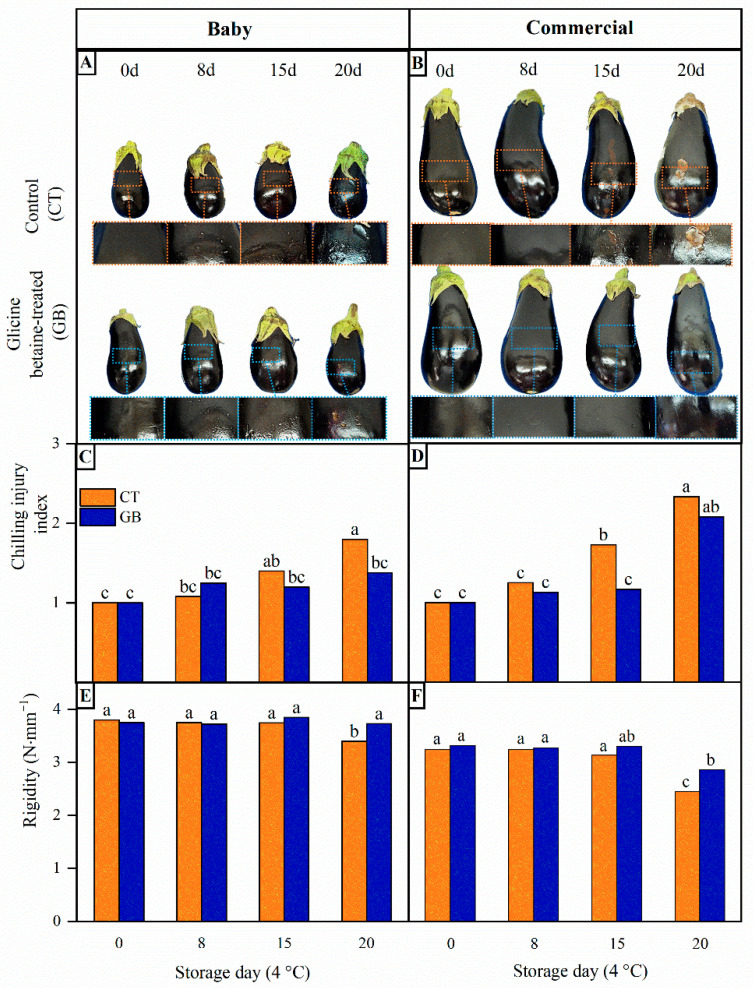
(**A**,**B**) Whole fruit appearance and a magnified view of chilling injury symptoms in the peel, (**C**,**D**) chilling injury index and (**E**,**F**) rigidity of control (CT) (**A**,**C**,**E**) and treated with 10 mM glycine betaine (GB) (**B**,**D**,**F**) eggplant fruits at baby and commercial maturity stage, during storage at 4 °C for 0, 8, 15 and 20 days. Different letters indicate significant differences between samples (*p* < 0.05).

Chilling injury in purple eggplant is first visible at the peel level. Initially, superficial pitting becomes evident, progressively increasing in size and severity over time and leading to peel scalding. In control fruit from both maturity stages, slight pitting was first observed at 8 d of storage. In BB control fruit, symptom progression remained relatively mild and only incipient scalds were detected at 20 d (Figure 2A). In contrast, CC control fruit exhibited more intense pitting, with visible scalds already apparent at 15 d and becoming more pronounced by 20 d, as shown in the magnified insets (Figure 2B). In addition, a progressive loss of brightness was observed during storage. These observations indicate that control CC fruit showed earlier and more pronounced pitting and scald compared to BB fruit, confirming higher susceptibility to CI. Immersion treatment with 10 mM glycine betaine effectively delayed the onset of symptoms in both BB and CC eggplants, postponing the appearance of pitting until 15 d of storage (Figure 2A,B). Moreover, scalds were almost absent in BB-treated fruit, while in CC fruit it became evident later, after 20 d.

These symptoms were quantified through chilling injury index (CII). In both BB and CC groups, the CII progressively increased throughout storage (Figure 2C,D). In control fruit, CC reached CII values that were 19 and 23% higher than BB after 15 and 20 d, indicating more pronounced damage. Additionally, CC control fruits exhibited a similar CII at 15 d to that observed in BB at 20 d. This confirms that CC eggplants exhibited greater cold sensitivity than more immature BB fruit, as previously reported in our work [12,53]. Notably, this behavior is distinctive for eggplants, being opposite to that observed in other *Solanaceous* fruits such as tomato and pepper, where immature fruits are typically more sensitive to chilling injury [11].

Our previous work demonstrated that GB treatment enhances chilling tolerance in commercially mature eggplants by improving fruit firmness, antioxidant capacity, and cell membrane integrity [13]. However, its effectiveness in baby eggplant has not been previously evaluated, and the response to GB may vary depending on the inherent sensitivity of the tissue to chilling stress. In the present study, GB revealed a clear protective effect in both maturity stages. GB treatment delayed the onset of chilling symptoms, resulting in reductions in the CII of 23% at 20 d in BB fruit and an even greater and earlier reduction, 32% at 15 d, in CC fruit compared with untreated controls (Figure 2C,D). Notably, the treatment was particularly effective in the more cold-sensitive fruit, CC fruit. The mitigation of chilling injury by GB has also been reported in other chilling-sensitive vegetables, such as sweet pepper [78] and zucchini [21], supporting its role as a promising postharvest strategy.

Fruit rigidity decreased during storage, particularly in control samples. CC fruit showed a 25% reduction at 20 d, whereas BB fruit decreased only by 11% (Figure 2E,F). In contrast, GB treatment effectively mitigated this loss of firmness, limiting the reduction to 14% in CC and only 5% in BB fruits, thereby preserving tissue integrity more efficiently. This suggests that GB treatment may help maintain structural integrity during cold storage by modulating cell wall metabolism [14].

Overall, these results indicate that BB fruit exhibited greater intrinsic tolerance to chilling stress, while GB treatment effectively enhanced resistance, particularly in the more sensitive CC fruit.

### 3.3. Chemometric Analysis of FTIR Spectra in GB-Treated Eggplant

**Figure 3:**
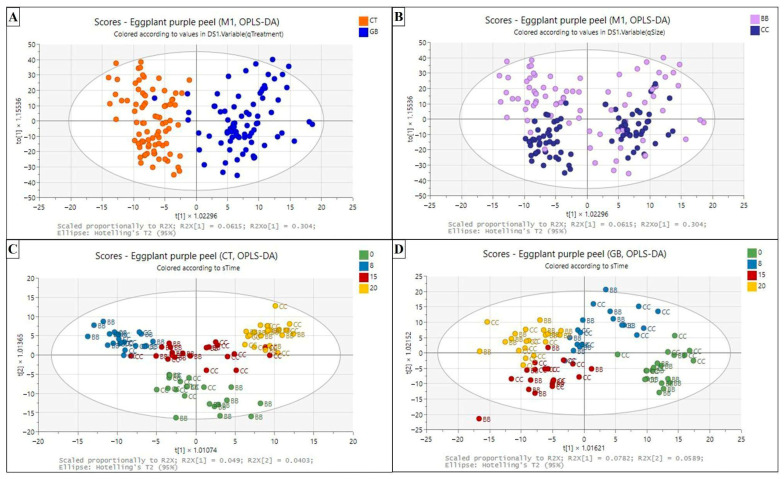
OPLS-DA of the spectral information for the eggplant fruit peels at baby (BB) and commercial (CC) maturity stages for the control (CT) and glycine betaine-treated (GB) groups during cold storage at 4 °C for 0, 8, 15 and 20 days. (**A**) CT and GB; (**B**) BB and CC eggplant peel, CT and GB; (**C**) CT eggplant peel at 0, 8, 15 and 20 days; (**D**) GB-treated eggplant peel at 0, 8, 15 and 20 days.

The FTIR spectral variations for the control (CT) and glycine betaine-treated (GB) eggplant peel at BB and CC stages were analyzed by means of OPLS-DA modeling (Figure 3). The OPLS-DA score plots revealed a clear separation of the samples according to treatment, maturity stage, and storage time. This indicated that these factors significantly influenced the FTIR spectral profiles and, therefore, the biochemical organization of the peel. The CT and GB samples were distinctly clustered in separate regions along the predictive component (Figure 3A), demonstrating that GB induced measurable changes in spectral profiles. The opposite distribution of both groups along the predictive axis suggested that the spectral variables contributing to the discrimination were inversely associated. In other words, the signals that increased in relative intensity for GB decreased for CT and vice versa. From a physiological point of view, this suggests that GB modulated the structural organization of the skin. Figure 3B uses the same underlying data as Figure 3A but highlights or colors the grouping by maturity stage (BB and CC). These scores exhibited a discernible trend toward separation. BB and CC fruit formed differentiated clusters, with BB samples showing greater dispersion, suggesting higher biochemical variability at early developmental stages. This pattern indicates that ontogenetic development contributes to the chemical structure of the peel and may influence its response to chilling conditions.

Time-dependent changes were evident in both CT and GB models. In the CT samples (Figure 3C), a progressive displacement from day 0 to day 20 was observed, reflecting cumulative biochemical modifications associated with cold storage and the onset of chilling injury. The shift across quadrants indicates directional changes in the spectral contributions of the specific molecular groups during storage. In contrast, GB-treated samples (Figure 3D) exhibited a more compact distribution at later storage stages (15 and 20 d), indicating stabilization of the biochemical state. This pattern indicates that the GB modulates the dynamics of cold-induced metabolic alterations, likely slowing the structural disorganization and maintaining the molecular integrity. Overall, the OPLS-DA models support the protective role of GB in preserving the peel structure under chilling stress. This confirms that the maturity stage strongly conditions the biochemical response of the eggplant fruit during postharvest storage.

### 3.4. FTIR Spectral Dynamics During Cold Storage

**Figure 4:**
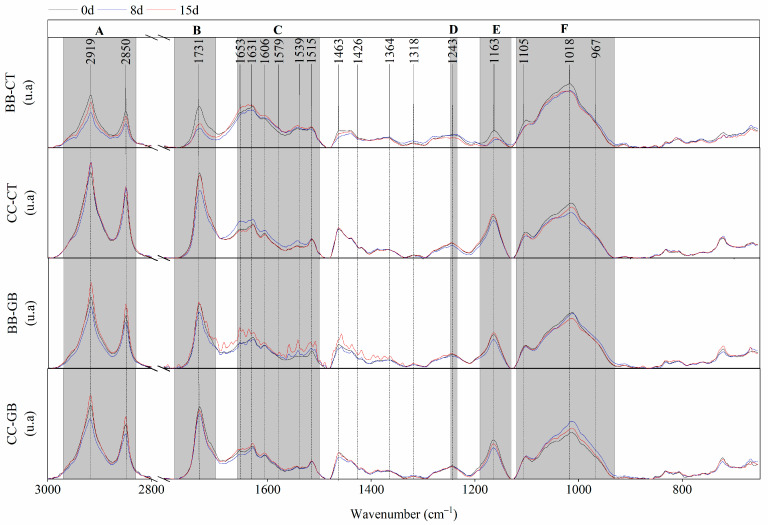
FTIR spectral information of the control group (CT) and treated (GB) peel of eggplant fruits at baby and commercial maturity stages, during storage for 0, 8 and 15 days at 4 °C. The highlighted or colored zones indicate characteristic FTIR signals from VIP scores.

Figure 4 shows the FTIR spectra of both BB and CC purple eggplant peel stored at chilling temperatures, both CT and GB fruits. The Variable Importance in Projection (VIP > 1) scores and contribution plots from the OPLS-DA models delineated the spectral biomarker pathway of the chilling injury in eggplant peel. These zones were gray colored in the figures to highlight their importance. The discriminant signals of the CT group from GB-treated samples were consistently associated with polysaccharides, polypeptide backbone of proteins, and phenolic compounds, which were described earlier. A further relevant contribution was observed at 967 cm^−1^, attributed to the strong symmetric stretching of the C–O–C bond that could be associated with the β-1,4-glycosidic bonds of cellulose. These bands reflect their central roles in the tissue reorganization under low temperature. As previously described, the biochemical alterations related to CI often precede the visible manifestation of superficial symptoms found on the peel. Therefore, although the FTIR spectra were analyzed for all days of storage, only those corresponding to days 0, 8, and 15 are shown here (Figure 4).

The FTIR analysis of the control eggplant peel revealed distinct temporal responses to chilling storage between BB (Figure 4, BB-CT) and CC fruit (Figure 4, CC-CT). Although both maturity stages exhibited biochemical alterations under low-temperature storage, their temporal patterns differed. In the C-H and CH_2_ aliphatic bands related to the lipid membrane-associated region (2919–2850 cm^−1^, colored zone A), BB-CT fruit showed a pronounced decrease at 8 d, followed by partial recovery at 15 d, whereas CC fruit remained relatively unchanged.

The ester C=O band in 1731–1733 cm^−1^ (colored zone B) progressively decreased in BB-CT fruit over time, indicating a probable continuous modification of pectin esterification. In contrast, CC fruit showed a transient decrease at 8 d followed by an increase at 15 d, probably due to oxidation of phenolic compounds to browning products [11,12].

The 1653–1515 cm^−1^ region (colored zone C), which includes Amide I and II vibrations as well as contributions from phenolic compounds (e.g., chlorogenic acid), anthocyanins (mainly delphinidin derivatives), and possibly glycoalkaloids, showed the most relevant differences. CC fruit showed early modulation at 8 d, whereas BB fruit exhibited delayed changes, becoming evident at 15 d. This suggests that CC fruit could undergo earlier metabolic and oxidative adjustments to chilling stress.

The vibration of heterocyclic rings at the band in 1243 cm^−1^ (colored zone D) related to flavonoids showed a similar trend: early decrease in CC-CT at 8 d and late decrease in BB-CT at 15 d, indicating both an earlier metabolic reorganization in CC fruit and that flavonoids were crucial familiar compounds in differences in CI sensitivity between eggplant stages.

The C-O-C bands associated with the glycosidic bond of flavonoids (1163 cm^−1^, colored zone E) and pyranose ring of polysaccharides (1105 cm^−1^, colored zone F), and C-O/C-C/C-OH bands associated with polysaccharides (wide region with peak at 1017 cm^−1^, colored zone F) [79] showed a progressive decrease in band intensity during storage in both maturity stages, reflecting structural alterations in cell wall components. However, these changes occurred earlier and more markedly in CC fruit.

Overall, the FTIR profiles indicated that while both maturity stages experienced membrane and cell wall perturbations during chilling, the CC fruit displayed earlier metabolic reorganization, especially at 8 d, whereas BB fruit exhibited sustained structural carbohydrate modifications. The bands in zones 1653–1515 (amide–phenolic, zone C), 1105 and 1017 (polysaccharide, zone F) cm^−1^ constitute clear spectral markers followed by 2919–2850 (lipid-associated, zone A), 1731 (ester, zone B), and 1243 (flavonoid-associated, zone D) cm^−1^ that distinguish the response of BB-CT and CC-CT fruits to chilling injury.

GB treatment delayed chilling injury and modified the FTIR profiles of both BB-GB (Figure 4, BB-GB) and CC-GB fruit (Figure 4, CC-GB). In the lipid-associated region (2919–2850 cm^−1^, zone A), both fruit types showed a decrease at 8 d followed by recovery at 15 d, indicating transient membrane perturbation. This pattern differed from CT fruit, where lipid signals either declined (BB) or remained unchanged (CC), suggesting that GB mitigates sustained lipid disorganization under chilling.

More pronounced stage-dependent differences were observed in the 1653–1515 cm^−1^ region (zone C), associated with protein amide vibrations and contributions from C–N-containing compounds (glycoalkaloids), phenolics, and anthocyanins. CC-GB fruit exhibited a slight increase at 15 d, unlike CC-CT, which increased earlier at 8 d. In BB-GB fruit, signal intensification at 15 d (particularly 1579–1515 cm^−1^) indicated a stronger and distinct metabolic response compared to both CC-GB and BB-CT, suggesting involvement of nitrogen-based compounds.

The polysaccharide-associated region (1017 cm^−1^, zone F) showed clear stage-specific responses. In BB-GB fruit, the band broadened at 8 d and decreased at 15 d, while in CC-GB fruit it increased markedly at 8 d and remained slightly elevated at 15 d. This contrasted with CC-CT fruit, where signals decreased throughout storage. These results suggest that GB more effectively modulates cell wall organization in chilling-sensitive (CC) fruit.

Overall, GB attenuated early metabolic shifts in the amide–phenolic region (zone C) and promoted stabilization of polysaccharide organization (zone F), supporting preservation of membrane–wall integrity and delayed structural degradation. This effect was more evident in CC fruit, where GB shifted the spectral response toward a delayed pattern resembling BB controls. Thus, the 1653–1515 and 1017 cm^−1^ regions emerge as reliable markers for chilling progression and GB-induced delay.

Phenolic–protein interactions may contribute to these spectral changes. Chlorogenic acid (CGA), abundant in eggplant peel, can form covalent and non-covalent complexes with proteins, altering their structure and functionality [80]. Additionally, anthocyanins interact with glycoproteins via non-covalent forces, enhancing signals in the 1500–1600 cm^−1^ region [81,82]. These interactions may explain the increased amide–phenolic signals, particularly in BB-GB fruit, suggesting that GB promotes associations between phenolic and nitrogen-based compounds, contributing to structural stabilization under chilling stress [80,83,84]. In parallel, anthocyanins can assemble non-covalently with glycoproteins via π–π stacking, hydrogen bonding, and hydrophobic interactions, stabilizing the flavylium chromophore and shifting spectral properties toward enhanced intensity in the 1500–1600 cm^−1^ region at highlighted zone C [81,82]. In line with the observed spectral signal increases, this behavior could indicate protein–phenolic associations as part of chilling injury onset, consistent with the stronger amide–phenolic signals observed in GB-BB peels, and suggesting that GB supplementation promotes phenolic and nitrogen-based compound interactions indirectly, amplifying the role of CGA and anthocyanins in modulating nitrogen-based conformation and their membrane-associated stabilization during chilling stress [80,83,84].

As a summary, the observed spectral differences in amide- and phenolic-associated regions may be consistent with GB-related changes in peel composition or molecular interactions. These results align with the known role of GB as an osmoprotectant and membrane stabilizer, which enhances cellular homeostasis under stress conditions [13]. Overall, GB treatment modulates the postharvest biochemical dynamics of eggplant peel in a size-dependent manner, with a stronger protective effect in larger fruits through stabilization of structural components and reduced compositional degradation. In addition, further studies including additional eggplant genotypes (e.g., striped and white) will be necessary to confirm the wider applicability of the observed effects.

## 4. Conclusions

This study demonstrates that fruit maturity stage is a key determinant of chilling injury susceptibility in eggplant, with commercially mature CC fruit showing earlier and more pronounced structural and physiological deterioration during cold storage. Pre-storage glycine betaine (GB) treatment effectively delayed symptom development and reduced chilling injury, particularly in the more sensitive fruit, CC.

FTIR spectroscopy combined with chemometric analysis enabled the identification of key spectral markers associated with chilling injury progression, mainly in the amide–phenolic (1653–1515 cm^−1^) and polysaccharide (~1017 cm^−1^) regions. The delayed modulation of these regions in GB-treated fruit indicates preservation of membrane–cell wall integrity. Importantly, this work provides the first comprehensive FTIR band assignment for eggplant peel, establishing a reference framework for future spectroscopic and postharvest studies in this species.

Overall, the use of FTIR and chemometrics appears a rapid, non-destructive, residue-free and robust approach to monitor postharvest metabolic and structural dynamics and evaluate the effectiveness of treatments aimed at reducing chilling injury in eggplant.

## Data Availability

The original contributions presented in this study are included in the article/Supplementary Material. Further inquiries can be directed to the corresponding author.

## Supplementary Materials

The following supporting information can be downloaded at: https://www.mdpi.com/article/10.3390/foods15101704/s1, Supplementary Material: Spectroscopic Profile of Phenolic and Nitrogen-Based Compounds.

## References

[1] Schudel S., Shoji K., Shrivastava C., Onwude D., Defraeye T. (2023). Solution Roadmap to Reduce Food Loss along Your Postharvest Supply Chain from Farm to Retail. Food Packag. Shelf Life.

[2] United Nations Environment Programme (2024). Food Waste Index Report 2024. Think Eat Save: Tracking Progress to Halve Global Food Waste.

[3] Wang S., Liu D., Sheng J., Wang S., Liu D., Sheng J. (2024). Prevention of Food Waste in China: Role and Impact of China’s Anti-Food Waste Law. Foods.

[4] Tsouvaltzis P., Babellahi F., Amodio M.L., Colelli G. (2020). Early Detection of Eggplant Fruit Stored at Chilling Temperature Using Different Non-Destructive Optical Techniques and Supervised Classification Algorithms. Postharvest Biol. Technol..

[5] Yuan Q., Jiang Y., Yang Q., Li W., Gan G., Cai L., Li W., Qin C., Yu C., Wang Y. (2024). Mechanisms and Control Measures of Low Temperature Storage-Induced Chilling Injury to Solanaceous Vegetables and Fruits. Front. Plant Sci..

[6] Kainat F., Ali M., Akbar A., Masih R., Mehnaz S., Sadiq M.B. (2023). Ultrasonic Extraction of Phenolic Compounds from Eggplant Peel and Formulation of Eggplant Peel Extract-Enriched Ice-Cream. J. Food Qual..

[7] Albornoz K., Zhou J., Yu J., Beckles D.M. (2022). Dissecting Postharvest Chilling Injury through Biotechnology. Curr. Opin. Biotechnol..

[8] Zhang P., Shao X., Wei Y., Xu F., Wang H. (2020). At-Harvest Fruit Maturity Affects Sucrose Metabolism during Cold Storage and Is Related to Chilling Injury in Peach. J. Food Sci. Technol..

[9] Qian C.-L., Mi H.-B., Zhao Y.-Y., He Z.-P., Mao L.-C. (2013). Effect of Maturity Stage on the Gene Expression of Antioxidative Enzymes in Cucumber (*Cucumis sativus* L.) Fruits Under Chilling Stress. J. Integr. Agric..

[10] Rai A., Kumari K., Vashistha P. (2022). Umbrella Review on Chilling Injuries: Post-Harvest Issue, Cause, and Treatment in Tomato. Sci. Hortic..

[11] Valenzuela J.L., Manzano S., Palma F., Carvajal F., Garrido D., Jamilena M. (2017). Oxidative Stress Associated with Chilling Injury in Immature Fruit: Postharvest Technological and Biotechnological Solutions. Int. J. Mol. Sci..

[12] Zaro M.J., Keunchkarian S., Chaves A.R., Vicente A.R., Concellón A. (2014). Changes in Bioactive Compounds and Response to Postharvest Storage Conditions in Purple Eggplants as Affected by Fruit Developmental Stage. Postharvest Biol. Technol..

[13] Guijarro M., Darré M., Careri L., Concellón A., Zaro M.J. (2025). Glycine Betaine Treatment Enhanced Eggplant Chilling Tolerance by Modulating Peel and Flesh Metabolic Responses. Horticulturae.

[14] Wu J., Tang R., Fan K. (2024). Recent Advances in Postharvest Technologies for Reducing Chilling Injury Symptoms of Fruits and Vegetables: A Review. Food Chem. X.

[15] Andrade-Cuvi M.J., Moreno C., Zaro M.J., Vicente A.R., Concellón A. (2017). Improvement of the Antioxidant Properties and Postharvest Life of Three Exotic Andean Fruits by UV-C Treatment. J. Food Qual..

[16] Li L., Kitazawa H., Zhang R., Wang X., Zhang L., Yu S., Li Y. (2019). New Insights into the Chilling Injury of Postharvest White Mushroom (Agaricus Bisporus) Related to Mitochondria and Electron Transport Pathway under High O_2_/CO_2_ Controlled Atmospheres. Postharvest Biol. Technol..

[17] Endo H., Miyazaki K., Ose K., Imahori Y. (2019). Hot Water Treatment to Alleviate Chilling Injury and Enhance Ascorbate-Glutathione Cycle in Sweet Pepper Fruit during Postharvest Cold Storage. Sci. Hortic..

[18] Loayza F.E., Brecht J.K., Simonne A.H., Plotto A., Baldwin E.A., Bai J., Lon-Kan E. (2021). A Brief Hot-Water Treatment Alleviates Chilling Injury Symptoms in Fresh Tomatoes. J. Sci. Food Agric..

[19] Zhang W., Jiang H., Cao J., Jiang W. (2021). Advances in Biochemical Mechanisms and Control Technologies to Treat Chilling Injury in Postharvest Fruits and Vegetables. Trends Food Sci. Technol..

[20] Zhang L., Huang Y., Sun F., Chen D., Netzel M., Smyth H.E., Sultanbawa Y., Deng Y., Fang M., Cozzolino D. (2021). The Effect of Maturity and Tissue on the Ability of Mid Infrared Spectroscopy to Predict the Geographical Origin of Banana (*Musa Cavendish*). Int. J. Food Sci. Technol..

[21] Yao W., Xu T., Farooq S.U., Jin P., Zheng Y. (2018). Glycine Betaine Treatment Alleviates Chilling Injury in Zucchini Fruit (*Cucurbita pepo* L.) by Modulating Antioxidant Enzymes and Membrane Fatty Acid Metabolism. Postharvest Biol. Technol..

[22] Razavi F., Mahmoudi R., Rabiei V., Aghdam M.S., Soleimani A. (2018). Glycine Betaine Treatment Attenuates Chilling Injury and Maintains Nutritional Quality of Hawthorn Fruit during Storage at Low Temperature. Sci. Hortic..

[23] Wang J., Lv M., He H., Jiang Y., Yang J., Ji S. (2020). Glycine Betaine Alleviated Peel Browning in Cold-Stored ‘Nanguo’ Pears during Shelf Life by Regulating Phenylpropanoid and Soluble Sugar Metabolisms. Sci. Hortic..

[24] Cebi N., Bekiroglu H., Erarslan A. (2023). Nondestructive Metabolomic Fingerprinting: FTIR, NIR and Raman Spectroscopy in Food Screening. Molecules.

[25] Zheng C., Li J., Liu H., Wang Y. (2024). Application of ATR-FTIR and FT-NIR Spectroscopy Coupled with Chemometrics for Species Identification and Quality Prediction of Boletes. Food Chem. X.

[26] Serna-Escolano V., Giménez M.J., Zapata P.J., Cubero S., Blasco J., Munera S. (2024). Non-Destructive Assessment of “Fino” Lemon Quality through Ripening Using NIRS and Chemometric Analysis. Postharvest Biol. Technol..

[27] Canteri M.H.G., Renard C.M.G.C., Le Bourvellec C., Bureau S. (2019). ATR-FTIR Spectroscopy to Determine Cell Wall Composition: Application on a Large Diversity of Fruits and Vegetables. Carbohydr. Polym..

[28] Amoriello T., Ciorba R., Ruggiero G., Masciola F., Scutaru D., Ciccoritti R. (2025). Vis/NIR Spectroscopy and Vis/NIR Hyperspectral Imaging for Non-Destructive Monitoring of Apricot Fruit Internal Quality with Machine Learning. Foods.

[29] Lukacs M., Vitalis F., Bardos A., Tormási J., Bec K.B., Grabska J., Gillay Z., Tömösközi-Farkas R.A., Abrankó L., Albanese D. (2024). Comparison of Multiple NIR Instruments for the Quantitative Evaluation of Grape Seed and Other Polyphenolic Extracts with High Chemical Similarities. Foods.

[30] Sachadyn-Król M., Budziak-Wieczorek I., Jackowska I. (2023). The Visibility of Changes in the Antioxidant Compound Profiles of Strawberry and Raspberry Fruits Subjected to Different Storage Conditions Using ATR-FTIR and Chemometrics. Antioxidants.

[31] Borges C.V., Amorim V.B.D.O., Ramlov F., Ledo C.A.D.S., Donato M., Maraschin M., Amorim E.P. (2014). Characterisation of Metabolic Profile of Banana Genotypes, Aiming at Biofortified Musa Spp. Cultivars. Food Chem..

[32] Massolo J.F., Concellón A., Chaves A.R., Vicente A.R. (2013). Use of 1-Methylcyclopropene to Complement Refrigeration and Ameliorate Chilling Injury Symptoms in Summer Squash. CyTA J. Food.

[33] Beatriz S., Directora M., Salvatori D.D. (2018). Desarrollo de Nuevos Productos Deshidratados a Partir de Peras (Var. Packham’s Triumph) de La Norpatagonia Argentina.

[34] Valerga L., Darré M., Zaro M.J., Arambarri A., Vicente A.R., Lemoine M.L., Concellón A. (2019). Micro-Structural and Quality Changes in Growing Dark-Purple Eggplant (*Solanum melongena* L.) as Affected by the Harvest Season. Sci. Hortic..

[35] Helilusiatiningsih N. (2021). Test of Chemical Compounds on Fruit Eggplant (*Solanum torvum*) Room Temperature Storage LCMS and FTIR Methods. Prosiding Seminar.

[36] Yan Z., Liang Y., Li Z., Lin D., Dou H., Li N., Yang Y. (2024). Wax Patterns, Textural Properties, and Quality Attributes of Two Eggplant (*Solanum melongena* L.) Cultivars during Storage. HortScience.

[37] Wang H., Nie Z., Wang T., Yang S., Zheng J. (2024). Comparative Transcriptome Analysis of Eggplant (*Solanum melongena* L.) Peels with Different Glossiness. Agronomy.

[38] Reynoud N., Geneix N., Petit J., D’Orlando A., Fanuel M., Marion D., Rothan C., Lahaye M., Bakan B. (2022). The Cutin Polymer Matrix Undergoes a Fine Architectural Tuning from Early Tomato Fruit Development to Ripening. Plant Physiol..

[39] Ingram G., Nawrath C. (2017). The Roles of the Cuticle in Plant Development: Organ Adhesions and Beyond. J. Exp. Bot..

[40] Bakan B., Marion D. (2017). Assembly of the Cutin Polyester: From Cells to Extracellular Cell Walls. Plants.

[41] Heredia-Guerrero J.A., Benítez J.J., Domínguez E., Bayer I.S., Cingolani R., Athanassiou A., Heredia A. (2014). Infrared and Raman Spectroscopic Features of Plant Cuticles: A Review. Front. Plant Sci..

[42] Reynoud N. (2023). Structure-Function Relationships of Plant Cuticle. Ph.D. Thesis.

[43] Sachadyn-Król M., Materska M., Chilczuk B., Karaś M., Jakubczyk A., Perucka I., Jackowska I. (2016). Ozone-Induced Changes in the Content of Bioactive Compounds and Enzyme Activity during Storage of Pepper Fruits. Food Chem..

[44] Girard A.L., Mounet F., Lemaire-Chamley M., Gaillard C., Elmorjani K., Vivancos J., Runavot J.L., Quemener B., Petit J., Germain V. (2012). Tomato GDSL1 Is Required for Cutin Deposition in the Fruit Cuticle. Plant Cell.

[45] Liu Y., Zhao J., Si M. (2025). Study on the distribution characteristics of α-Solanine in different parts of Solanum nigrum based on infrared spectroscopy. Results Chem..

[46] Montagni T., Rodríguez Chialanza M., Cerdá M.F. (2023). Blueberries as a Source of Energy: Physical Chemistry Characterization of Their Anthocyanins as Dye-Sensitized Solar Cells’ Sensitizers. Solar.

[47] Skolik P., Morais C.L.M., Martin F.L., McAinsh M.R. (2019). Determination of Developmental and Ripening Stages of Whole Tomato Fruit Using Portable Infrared Spectroscopy and Chemometrics. BMC Plant Biol..

[48] Río Segade S., Paissoni M.A., Giacosa S., Bautista-Ortín A.B., Gómez-Plaza E., Gerbi V., Rolle L. (2019). Winegrapes Dehydration under Ozone-Enriched Atmosphere: Influence on Berry Skin Phenols Release, Cell Wall Composition and Mechanical Properties. Food Chem..

[49] Lai X., Khanal B.P., Knoche M. (2016). Mismatch between Cuticle Deposition and Area Expansion in Fruit Skins Allows Potentially Catastrophic Buildup of Elastic Strain. Planta.

[50] Bauer S., Schulte E., Thier H.P. (2005). Composition of the Surface Waxes from Bell Pepper and Eggplant. Eur. Food Res. Technol..

[51] Bauer S., Schulte E., Thier H.P. (2004). Composition of the Surface Wax from Tomatoes. Eur. Food Res. Technol..

[52] Darré M., Valerga L., Zaro M.J., Lemoine M.L., Concellón A., Vicente A.R. (2022). Low temperature conditioning improves American eggplant (*Solanum melongena* L.) storage compatibility. J. Hortic. Sci. Bio-Technol..

[53] Sánchez-Hernández E., González-García V., Palacio-Bielsa A., Lorenzo-Vidal B., Buzón-Durán L., Martín-Gil J., Martín-Ramos P. (2023). Antibacterial Activity of Ginkgo Biloba Extracts against Clavibacter Michiganensis Subsp. Michiganensis, Pseudomonas Spp., and Xanthomonas Vesicatoria. Horticulturae.

[54] Benítez J.J., Guzmán-Puyol S., Vilaplana F., Heredia-Guerrero J.A., Domínguez E., Heredia A. (2021). Mechanical Performances of Isolated Cuticles Along Tomato Fruit Growth and Ripening. Front. Plant Sci..

[55] Szymanska-Chargot M., Zdunek A. (2013). Use of FT-IR Spectra and PCA to the Bulk Characterization of Cell Wall Residues of Fruits and Vegetables Along a Fraction Process. Food Biophys..

[56] Liu X., Renard C.M.G.C., Bureau S., Le Bourvellec C. (2021). Revisiting the Contribution of ATR-FTIR Spectroscopy to Characterize Plant Cell Wall Polysaccharides. Carbohydr. Polym..

[57] Domínguez E., Cuartero J., Heredia A. (2011). An Overview on Plant Cuticle Biomechanics. Plant Sci..

[58] Khanal B.P., Knoche M. (2017). Mechanical Properties of Cuticles and Their Primary Determinants. J. Exp. Bot..

[59] Raba D.N., Poiana M.A., Borozan A.B., Stef M., Radu F., Popa M.V. (2015). Investigation on Crude and High-Temperature Heated Coffee Oil by ATR-FTIR Spectroscopy along with Antioxidant and Antimicrobial Properties. PLoS ONE.

[60] Wang L., Gong H., Peng N., Zhang J.Z. (2018). Molecular Adsorption Mechanism of Elemental Carbon Particles on Leaf Surface. Environ. Sci. Technol..

[61] Kazemi M., Khodaiyan F., Hosseini S.S. (2019). Eggplant Peel as a High Potential Source of High Methylated Pectin: Ultrasonic Extraction Optimization and Characterization. LWT.

[62] Domínguez-Pérez L.A., Lagunes-Gálvez L.M., Barajas-Fernández J., de los Ángeles Olán-Acosta M., García-Alamilla R., García-Alamilla P. (2019). Caracterización Vibracional de Grupos Funcionales En Granos de Cacao Durante El Tostado Usando Espectroscopía de Infrarrojo Por Transformada de Fourier. Acta Univ..

[63] Derksen G.C.H., Blommaert L., Bastiaens L., Hasşerbetçi C., Fremouw R., van Groenigen J., Twijnstra R.H., Timmermans K.R. (2023). ATR-FTIR Spectroscopy Combined with Multivariate Analysis as a Rapid Tool to Infer the Biochemical Composition of Ulva Laetevirens (Chlorophyta). Front. Mar. Sci..

[64] Castillo-Guaca S.M., Muñoz-Pabon K.S., Bravo-Gómez J.E., Roa-Acosta D.F., Vergara Escobar J.F. (2023). Identification of Macronutrients by FT-IR Analysis and Physicochemical Characterization of Snacks Elaborated from Quinoa (Chenopodium Quinoa Willd) and Sacha Inchi (Plukenetia Volubilis). F1000Research.

[65] Lee C.M., Mohamed N.M.A., Watts H.D., Kubicki J.D., Kim S.H. (2013). Sum-Frequency-Generation Vibration Spectroscopy and Density Functional Theory Calculations with Dispersion Corrections (DFT-D2) for Cellulose Iα and Iβ. J. Phys. Chem. B.

[66] Fahey L.M., Nieuwoudt M.K., Harris P.J. (2017). Predicting the Cell-Wall Compositions of *Pinus radiata* (Radiata Pine) Wood Using ATR and Transmission FTIR Spectroscopies. Cellulose.

[67] Vlachos N., Skopelitis Y., Psaroudaki M., Konstantinidou V., Chatzilazarou A., Tegou E. (2006). Applications of Fourier Transform-Infrared Spectroscopy to Edible Oils. Anal. Chim. Acta.

[68] Christou C., Agapiou A., Kokkinofta R. (2018). Use of FTIR Spectroscopy and Chemometrics for the Classification of Carobs Origin. J. Adv. Res..

[69] Krysa M., Szymańska-Chargot M., Zdunek A. (2022). FT-IR and FT-Raman Fingerprints of Flavonoids—A Review. Food Chem..

[70] Stepanovich E.Y., Aliyev P.N. (2023). Analysis of Cyanidin, Delphinidin, Malvidin and Pelargonidin Anthocyanins in Food Products by Infrared Spectroscopy. Ecol. Bull. Res. Cent. Black Sea Econ. Coop..

[71] Bhushan B., Bibwe B., Pal A., Mahawar M.K., Dagla M.C., KR Y., Jat B.S., Kumar P., Aggarwal S.K., Singh A. (2023). FTIR Spectra, Antioxidant Capacity and Degradation Kinetics of Maize Anthocyanin Extract under Variable Process Conditions. Appl. Food Res..

[72] Mukhametov A., Mamayeva L., Kazhymurat A., Akhlan T., Yerbulekova M. (2023). Study of Vegetable Oils and Their Blends Using Infrared Reflectance Spectroscopy and Refractometry. Food Chem. X.

[73] Liang N., Lu X., Hu Y., Kitts D.D. (2016). Application of Attenuated Total Reflectance–Fourier Transformed Infrared (ATR-FTIR) Spectroscopy To Determine the Chlorogenic Acid Isomer Profile and Antioxidant Capacity of Coffee Beans. J. Agric. Food Chem..

[74] Paraíso C.M., dos Santos S.S., Ogawa C.Y.L., Sato F., dos Santos O.A.A., Madrona G.S. (2020). *Hibiscus sabdariffa* L. Extract: Characterization (FTIR-ATR), Storage Stability and Food Application. Emir. J. Food Agric..

[75] Cervantes-Güicho V.D.J., Ríos-González L.J., Reyes-Alvarado A.G., Morales-Martínez T.K. (2024). Microwave-Assisted Extraction of Flavonoids from Lechuguilla Guishe. Ing. Agrícola Biosist..

[76] Chylińska M., Szymańska-Chargot M., Zdunek A. (2016). FT-IR and FT-Raman Characterization of Non-Cellulosic Polysaccharides Fractions Isolated from Plant Cell Wall. Carbohydr. Polym..

[77] Zineb A.B., Faissal K., Hassan A., Rachid H., Abou B., Kacem E., Mina Q., Mounir O., Achaby E., Bahloul A. (2021). Characteristics of Cellulose Microfibers and Nanocrystals Isolated from Doum Tree (*Chamaerops humilis* Var. Argentea). Cellulose.

[78] Wang Q., Ding T., Zuo J., Gao L., Fan L. (2016). Amelioration of Postharvest Chilling Injury in Sweet Pepper by Glycine Betaine. Postharvest Biol. Technol..

[79] Wang J., Wang J., Hao J., Jiang M., Zhao C., Fan Z. (2024). Antioxidant Activity and Structural Characterization of Anthocyanin–Polysaccharide Complexes from Aronia Melanocarpa. Int. J. Mol. Sci..

[80] Tarahi M., Gharagozlou M., Niakousari M., Hedayati S. (2024). Protein–Chlorogenic Acid Interactions: Mechanisms, Characteristics, and Potential Food Applications. Antioxidants.

[81] Xing C., Chen P., Zhang L. (2023). Computational Insight into Stability-Enhanced Systems of Anthocyanin with Protein/Peptide. Food Chem. Mol. Sci..

[82] Zhang R., Ye S., Guo Y., Wu M., Jiang S., He J. (2023). Studies on the Interaction between Homological Proteins and Anthocyanins from Purple Sweet Potato (PSP): Structural Characterization, Binding Mechanism and Stability. Food Chem..

[83] Shi C., Guo C., Wang S., Li W., Zhang X., Lu S., Ning C., Tan C. (2024). The Mechanism of Pectin in Improving Anthocyanin Stability and the Application Progress of Their Complexes: A Review. Food Chem. X.

[84] Yang Y., Chen L., Chen M., Liu F., Zhong F. (2025). Interactions between Rice Protein and Anthocyanin with Different PH-Cycle: Structural Characterization, Binding Mechanism and Stability. Food Hydrocoll..

