# Supplementary material for: Effect of Glycine Betaine on Chilling Injury in Eggplant Peel Revealed by FTIR Spectroscopy
Source: Foods. 2026 May 13;15(10):1704. doi: 10.3390/foods15101704 (PMC13205402; doi:10.3390/foods15101704)
Supplement: Supplementary file 1 [file foods-15-01704-s001.zip › foods-4262712-supplementary.pdf]

Supplementary information for Effect of Glycine Betaine on Chilling Injury in Eggplant Peel Revealed by FT-IR Spectroscopy.

Michelle Guijarro[1], María José Zaro[1], Analía Concellón[1], Gemma Montalvo[2], Fernando Ortega[3], Armando Echeverría[4], Pablo Moncayo[5], and Luis Ramos-Guerrero[6]*

Supplementary 1

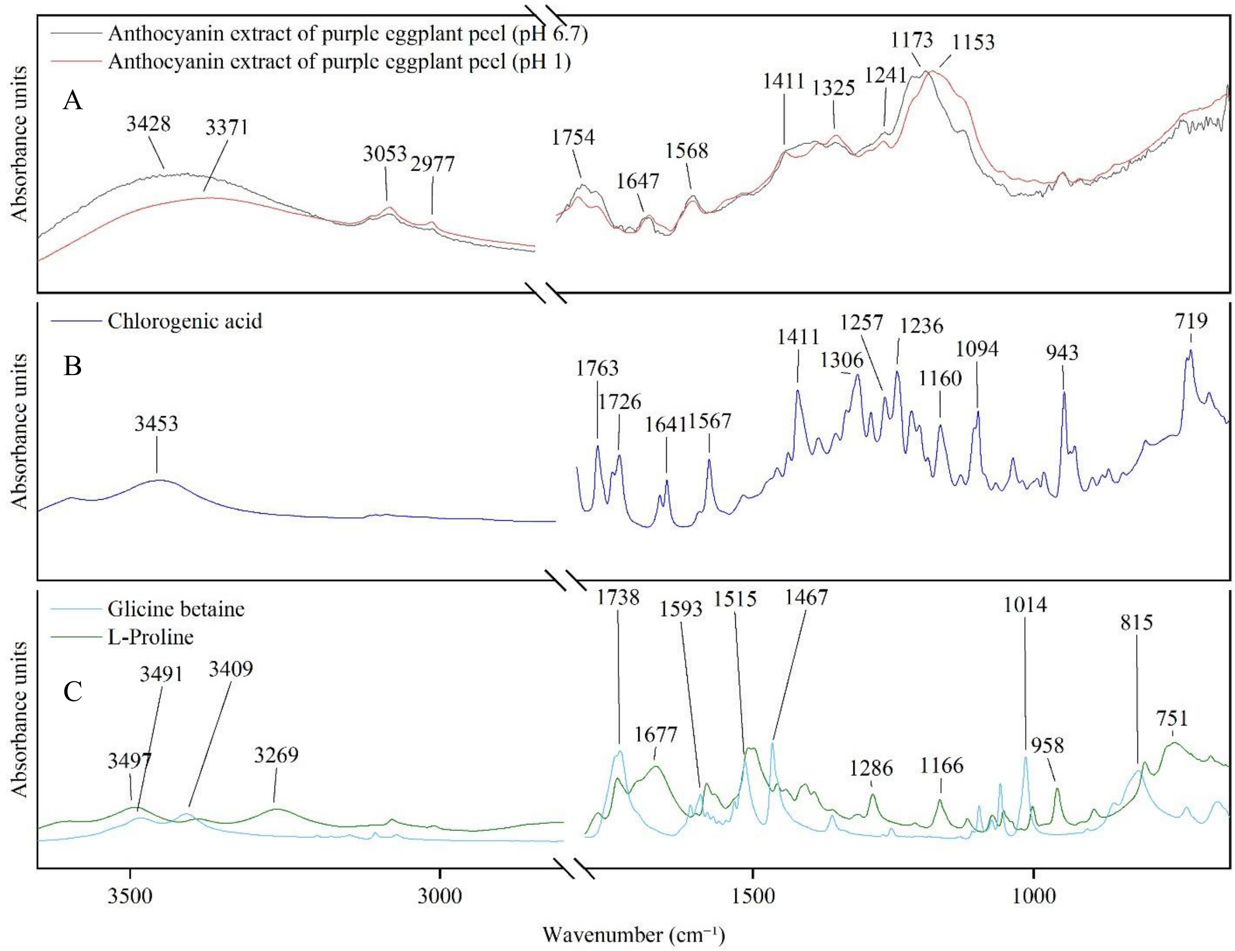

Figure S1. FTIR spectra of compounds of interest to assignation process, such as anthocyanin extracted from eggplant peel in different pH (A), standard of chlorogenic acid (B) and standards of nitrogen-based compounds (C).

All spectra were collected by co-addition of 64 scans at a resolution of 4 cm^{-1} in the range of 4000–600 cm^{-1}. Each spectrum was ratioed against a background spectrum obtained every 15 minutes, these spectrums were performed using a NicoletTM iSTM10 (Thermo Scientific, Madison, USA) device.
